# Ovarian aging, cardiovascular risk and inflammation: insights from the OVA study

**DOI:** 10.1186/s13048-025-01754-8

**Published:** 2025-07-26

**Authors:** C. Verhaeghe, KJ. Lindquist, ME. Bleil, M. Rosen, RF. Redberg, D. Haisenleder, CE. McCulloch, Marcelle I. Cedars

**Affiliations:** 1 Department of Obstetrics, Gynecology, and Reproductive Sciences, University of California, 499 Illinois St., 6th Floor, Box 0916, San Francisco, CA 94158 USA; 2 Department of Epidemiology and Biostatistics, University of California, San Francisco, CA USA; 3 Child, Family, and Population Health Nursing, School of Nursing, University of Washington, Seattle, WA 98195 USA; 4 Philip R Lee Institute for Health Policy Studies, San Francisco, CA USA; 5 Department of Medicine, UCSF School of Medicine, San Francisco, CA USA; 6 UVA Center for Research in Reproduction, Ligand Core, University of Virginia, Charlottesville, VA USA

**Keywords:** Ovarian aging, Chronic inflammation, Inflammatory biomarkers, anti-Müllerian hormone, Antral follicle count, Metabolic syndrome, Cardiovascular risk.

## Abstract

**Background:**

Cardiovascular disease is the leading cause of death among women and is associated with both metabolic syndrome and ovarian aging. Chronic inflammation has been proposed as a potential common underlying mechanism linking these conditions. This study aimed to examine the associations between inflammatory markers (interleukin-6, tumor necrosis factor-alpha, high-sensitivity C-reactive protein) and metabolic syndrome, with markers of ovarian aging and cardiovascular risk.

**Results:**

In the cross-sectional analysis of 829 women aged 25–45, no significant associations were found between inflammatory markers, metabolic syndrome, and ovarian aging measures (anti-Müllerian hormone [AMH] and antral follicle count [AFC]), except for a modest association between metabolic syndrome and AMH (mean difference 0.085; 95% CI: 0.035 to 0.134). Similarly, inflammatory markers and metabolic syndrome were not significantly associated with the Framingham Risk Score. In the longitudinal analysis of 307 participants, changes in AMH and AFC were not associated with inflammatory markers or metabolic syndrome. However, higher levels of IL-6 and TNF-α were associated with the Framingham Risk Score, whereas hsCRP and metabolic syndrome were not.

**Conclusion:**

These findings do not support the hypothesis that inflammation is a central mechanism linking ovarian aging to cardiovascular risk. The absence of consistent associations across analyses suggests that alternative pathways may underlie this relationship. Further research incorporating a broader range of biomarkers is warranted to elucidate the complex interactions between reproductive aging and cardiovascular health in women.

**Supplementary Information:**

The online version contains supplementary material available at 10.1186/s13048-025-01754-8.

## Background

Cardiovascular disease (CVD) is the leading cause of mortality among women, accounting for 48.1% of total deaths in 2019 [1]. Ovarian aging, as a marker of oocyte quantity through the reproductive years, and CVD share several biological mechanisms, and research has shown links between early menopause, diminished ovarian reserve, and increased cardiovascular risk [2]. As well, menopausal status is a female-specific CVD risk factor [3]. Chronic inflammation plays a key role in the development of CVD [4–7] and could also be a critical factor in ovarian aging [8–13].

Ovarian aging involves both quantitative and qualitative changes in the ovarian oocyte reserve [14]. The best markers for assessing ovarian reserve are Anti-Müllerian Hormone (AMH) and Antral Follicle Count (AFC) [15]both of which evaluate only quantitative decline. Factors affecting decline in ovarian reserve include epigenetic regulation, DNA damage, genetic mutations, telomere attrition, oxidative stress, and an inflammaging environment [8–10, 13].

Chronic inflammation is not only implicated in CVD but is also a central element of metabolic syndrome, a cluster of metabolic conditions that doubles the risk of developing CVD within the next 5 to 10 years [1]. Metabolic syndrome is itself associated with increased inflammatory activity [16]making it a potential marker of chronic inflammation and an indicator of risk for both CVD and ovarian aging [17].

Although connections have been suggested between inflammation, metabolic syndrome, ovarian aging, and CVD, the precise nature of these relationships remains underexplored [18]. Our study aims to investigate inflammation as a key mechanism linking ovarian aging and CVD. We therefore evaluated the inflammatory markers IL-6, TNF-α, hsCRP, and metabolic syndrome as potential indicators of ovarian aging and CVD. To achieve this, we conducted a cross-sectional analysis followed by a longitudinal study within a large cohort of healthy women.

## Materials and methods

### Participants

The cross-sectional analysis utilized data from the Ovarian Aging cohort (OVA) study, while the longitudinal analysis was based on the Ovarian Aging and Cardiovascular Risk cohort study (OVA.CV), an extension of the original OVA study. Details regarding the OVA study have been previously published [19, 20]. This study utilizes a cohort recruited through Kaiser Permanente (KP) of Northern California. The KP cohort reflects the general population in terms of socio-demographic and health characteristics, particularly in comparison to other insured populations [21].

Baseline selection criteria were identical to those of the OVA study: participants were women aged between 25 and 45, with regular menstrual cycles, and with their uterus and both ovaries intact. All participants were required to self-identify their race/ethnicity with both parents of the same racial/ethnic group. Exclusion criteria in the OVA study included reporting a major medical illness, taking medications affecting the menstrual cycle within the 3 months prior to study participation, or being currently pregnant or breastfeeding.

Participants recruited, for longitudinal follow-up, initially attended an OVA.CV visit, with a subset returning for a second visit at least two years later. Consequently, some women had one OVA.CV visit while others had two. Visits were scheduled between 8 and 16 years after their participation at baseline. Visits occurred on menstrual cycle days 2–4 for those cycle regularly cycling. Participants who recently had a cyst/fibroid removal, miscarriage, or recently completed pregnancy or breastfeeding waited at least 3 months before the visit. Women reporting no periods or very irregular cycles are seen at random times. Those on combined hormonal contraceptives were required to discontinue use for 2 months before the visit. Both the OVA study protocol and the OVA.CV protocol included an in-person interview, anthropometric assessment, transvaginal ultrasound (TVUS) on menstrual cycle days 2–4, and fasting blood and urine collection.

**Fig. 1:**
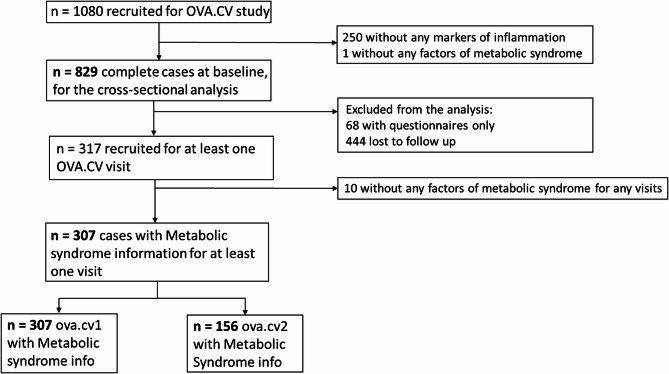
Flow diagram

We excluded women who lacked markers for inflammation (interleukin-6 (IL-6), Tumor Necrosis Factor alpha (TNF-alpha) and high-sensitivity C-Reactive Protein (hsCRP)), and information on metabolic syndrome (presence or absence) at baseline. Then subjects were excluded if components of metabolic syndrome were missing for OVA.CV visits. (Fig. 1).

The study protocol was approved by the University of California, San Francisco Committee on Human Research, written consent was obtained from all study participants.

### Measures

#### Ovarian aging

AFC was evaluated using transvaginal ultrasound with a 5-8mHz probe on menstrual cycle days 2–4. Follicles were identified as echo-free structures in the ovaries, measuring between 2 and 10 mm in average diameter across two dimensions [11]. Each follicle measurement was repeated, and the mean value was used. The total follicle count across both ovaries was aggregated to determine the AFC.

AMH levels were obtained through blood samples collected during the same window of their menstrual cycle, or randomly for those without regular cycles, and analyzed using the 2-site ELISA (picoAMH ELISA, Ansh Labs). All sample were run in batch through the Ligand Assay and Analysis Core Laboratory at the University of Virginia (intra-assay variance = 1.3; inter-assay variance = 6.7, lower limit of detection = 12 pg/mL).

Both AFC and AMH values were treated as continuous variables for analysis and log-transformed to meet normal distribution assumptions.

### Inflammation

Baseline measurements included the collection of IL-6, TNF-alpha and hsCRP from each participant. IL-6 and TNF-alpha were analyzed using the Quantikine High Sensitivity ELISA test, while hsCRP was measured with the IMMULITE 2000 High Sensitivity CRP assay (analytical sensitivity of 0.1 mg/L and a reportable range of 0.2 to 100 mg/L.). All inflammatory markers were obtained from blood samples taken during the same phase of each participant’s menstrual cycle. These markers were all analyzed as continuous variables and a log-transformation was used for the analysis.

### Cardiovascular risk factors

Parameters of cardio-metabolic health were selected to represent the individual components of metabolic syndrome (triglycerides, HDL, Fasting glucose, waist circumference, and hypertension) [22]. Assays for triglycerides, HDL, and fasting glucose were performed by Quest Diagnostics (San Jose, CA). Lipids were assayed using enzymatic methods (triglycerides: intraassay coefficient of variation [CV] was 1.99–3.45%; HDL: intraassay CV was 1.15–2.02%). Fasting glucose was assayed by the glucose oxidase method (intraassay CV: 1.25–2.00%).

Each risk factor was categorized as binary (yes/no) based on established clinical thresholds: (1) Fasting glucose (Yes if ≥ 100 mg/dL or the presence of type 2 diabetes as per questionnaire); (2) Triglycerides (Yes if ≥ 150 mg/dL); (3) HDL cholesterol (Yes if < 50 mg/dL); (4) Waist circumference (Yes if > 88 cm, or > 80 cm for Chinese participants); (5) Blood pressure (Yes for a history of hypertension, use of anti-hypertensive medication, or a reading ≥ 130/85 mmHg). Metabolic syndrome was determined based on the presence of at least three of these risk factors (Yes = 1, No = 0) [22].

The Framingham Risk Score was calculated to estimate the 10-year cardiovascular risk for participants at each follow-up visit using the `frisk’ package in R [23]. As systolic blood pressure measurements were not available at baseline, we used coefficients from a linear regression model derived from follow-up data and other individual components of the score (age, cholesterol levels, HDL cholesterol, smoking status, diabetes, and BMI) to estimate the score at baseline. Framingham scores were treated as a continuous outcome variable.

### Other variables

We used on a range of variables derived from the questionnaire. For cigarette smoking, participants were classified as either current/past smokers or never smokers. Race/ethnicity was considered for all analyses. Education level was categorized broadly from less than high school education to advanced degrees (less than high school or some high school education, high school graduate or equivalent (GED), some college or associate degree/vocational training, college graduate, graduate school (PhD, MS), and professional degrees (MD, JD, DDS, MBA)).Parity was assessed based on the number of live births (having one or more live births or none).Household income levels were segmented into detailed ranges: below $5,000; $5,000–$15,999; $16,000–$24,999; $25,000–$34,999; $35,000–$49,999; $50,000–$74,999; $75,000–$99,999; and over $100,000. Body Mass Index (BMI) was calculated from each participant’s weight in kilograms divided by their height in meters squared (kg/m²). And finally, age at menarche was self-reported and treated as a continuous variable.

### Statistical analysis

First, we performed a cross sectional analysis at baseline, to explore the association between inflammation, ovarian aging and cardiovascular risk. Linear regression for continuous variables was performed. Analyses were initially adjusted for age and BMI. The fully adjusted model was also adjusted for smoking status, income, education levels, race, parity, and menarche age. Income and educational levels were treated as ordinal variables; age, BMI, and menarche age as continuous variables; and parity, race, and smoking status as categorical variables. Age is the strongest predictor of both AMH and AFC [24, 25] and BMI is known to impact AMH levels [26]. The adjustment variables, for the fully adjusted model were chosen a-priori based on prior evidence that they might confound the association [27–32].

We conducted longitudinal analyses using linear generalized estimating equation (GEE) with robust variance estimates to account for the fact that some participants had multiple follow-up measurements. Inverse probability weighting was used to account for incomplete outcome data [33]. Probability weights were calculated using a binomial logistic regression model where the outcome was an indicator of missing outcome data at a follow-up visit, given the previous visit’s outcome value and all covariates included in the fully adjusted models including follow-up time, baseline inflammatory marker values, age, race/ethnicity, income, education, age at menarche, BMI, parity and tobacco use. Ovarian aging was assessed by subtracting values at the OVA.CV visit from baseline and applying a natural log transformation. Coefficients from GEE models are expressed as the difference in AMH or AFC decline or Framingham risk scores per one standard deviation increase in each inflammatory marker. Analyses investigating the relationships between baseline inflammation markers, ovarian aging and Framingham risk score were adjusted as noted above, in addition to adjusting for years between baseline and each visit and for baseline AMH, AFC, and Framingham scores for each respective outcome.

Lastly because of the number of participants that were excluded from the analysis between baseline (OVA) and follow-up (OVA.CV), we compared OVA.CV cohort to the OVA. Due to the attrition rate, a sample size calculation was performed assuming 80% power and the range of observed effect sizes to determine whether our sample size resulted in an underpowered test for association in the GEE models.

All associations were tested at a nominal level of 0.05 with no correction for multiple testing.

Analyses were performed using R statistical software version 4.4.0 [34].

## Results

### Population description

At baseline, 1,080 patients were enrolled. Of these, 250 were excluded due to the absence of inflammation markers, and one patient was excluded due to the absence of markers for metabolic syndrome. Consequently, for the cross-sectional analysis at baseline, 829 patients were analyzed.

Of these 829 patients with complete information at baseline, 317 were returned for the OVA.CV visits, while 512 agreed to questionnaire only (68) or were lost to follow-up (444). After excluding 10 women without factors for defining metabolic syndrome, the longitudinal analysis was based on 307 participants (Fig. 1).

### Sample characteristics at baseline (*n* = 829)

By design, the mean age was 35.2 years (SD = 5.5). The racial/ethnic composition was 30.5% Caucasian, 24.2% African American, 23.4% Latina, 18% Chinese, and 3.9% Filipino. Regarding parity, 57.4% were nulliparas. The mean BMI was 27.5 (SD = 6.97). Regarding cardiometabolic factors, 10.4% met the criteria for metabolic syndrome. Concerning ovarian aging, the median AFC was 14.0 (IQR= [8.0, 20.8]), and the AMH level was 3.5 ng/mL (IQR = 1.6, 6.3). Finally, regarding inflammatory markers, the median IL6 level was 1.7 pg/mL (IQR = 1.0, 2.9) the, median TNF-alpha level was 0.3 pg/mL (IQR = 0.2–0.5), and the median hsCRP level was 1.7 mg/L (IQR = 0.7–4.6) (Table 1).

Among those 829 patients, 307 had at least one follow-up. At baseline, for those 307 women, the mean age was 35.8 years (SD = 5.43). The racial/ethnic composition was 31.6% Caucasian, 22.5% African American, 22.1% Latina, 20.8% Chinese, and 2.9% Filipino. The mean BMI was 27.4 (SD = 7.18). As for cardiometabolic factors, 10.1% met the criteria for metabolic syndrome at baseline. The median AFC was 13.0 (IQR=[8.0, 21.0]) and the median AMH level was 3.4 ng/mL (IQR = 1.6, 6.1). Finally, the median IL-6 level was 1.6 pg/mL (IQR = 0.9, 2.5), the median TNF-alpha level was 0.3 pg/mL (IQR = 0.2–0.5), and the median hsCRP level was 1.7 mg/L (IQR = 0.7–4.3) (Table 1).

For patient demographics at follow-up visits 1 and 2, please refer to Table 1.

**Table 1:** Characteristics of participants in Cross-Sectional and longitudinal analyses

	Cross-Sectional cohort	Longitudinal study (OVA.CV) Baseline	Longitudinal cohort (OVA.CV) Visit 1		Longitudinal cohort (OVA.CV) Visit 2	
	829	307	307		156	
**Age (mean (SD))**	35.2 (5.5)	35.8 (5.4)	47.3 (5.8)		48.9 (5.8)	
**BMI (mean (SD))**	27.5 (7.0)	27.4 (7.2)	28.9 (7.2)		28.5 (6.9)	
**Waist Circumference (median [IQR])**	80.3 [72.4, 93.1]	80.3 [72.4, 92.2]	91.4 [82.6, 104.1]		88.9 [81.3, 102.9]	
**Nulliparity (%)**	476 (57.4)	172 (56.0)	142 (46.3)		85 (54.5)	
**Race (%)**						
Caucasian	253 (30.5)	97 (31.6)	97 (31.6)		61 (39.1)	
African American	201 (24.2)	69 (22.5)	69 (22.5)		28 (17.9)	
Chinese	149 (18.0)	64 (20.8)	64 (20.8)		37 (23.7)	
Filipino	32 (3.9)	9 (2.9)	9 (2.9)		3 (1.9)	
Latina	194 (23.4)	68 (22.1)	68 (22.1)		27 (17.3)	
**Income (%)**						
Less than $5,000	8 (1.0)	5 (1.6)	1 (0.4)		2 (1.4)	
$5,000 through $11,999	17 (2.1)	6 (2.0)	6 (2.2)		1 (0.7)	
$12,000 through $15,999	21 (2.5)	7 (2.3)	2 (0.7)		0 (0.0)	
$16,000 through $24,999	51 (6.2)	16 (5.2)	8 (2.9)		3 (2.1)	
$25,000 through $34,999	105 (12.7)	32 (10.5)	10 (3.6)		6 (4.2)	
$35,000 through $49,999	181 (22)	66 (21.6)	26 (9.4)		8 (5.6)	
$50,000 through $74,999	176 (21.4)	80 (26.2)	43 (15.5)		21 (14.8)	
$75,000 through $99,999	102 (12.4)	36 (12.1)	52 (18.8)		24 (16.9)	
$100,000 and greater	163 (19.8)	56 (18.4)	129 (46.6)		77 (54.2)	
**Education Level (%)**						
Less than high school / Some high school	67 (8.1)	18 (5.9)	16 (5.4)		2 (1.4)	
High school graduate/GED	82 (9.9)	26 (8.5)	29 (9.8)		7 (4.8)	
Some college (AA) /Vocational/technical school	208 (25.1)	85 (27.8)	66 (22.3)		31 (21.1)	
College graduate (Bachelor’s degree)	307 (37.0)	109 (35.6)	96 (32.4)		49 (33.3)	
Graduate school (Doctorate, Master’s degree)/ Professional school (MD, JD, DDS, MBA)	165 (19.9)	69 (22.2)	89 (30.1)		58 (39.5)	
**Smoking Status (%)**	207 (25.0)	72 (23.5)	51 (17.8)		25 (17.6)	
**Pathological Triglycerides (%)**	93 (11.2)	26 (8.5)	43 (14.1)		26 (19.0)	
**Pathological Fasting Glucose (%)**	59 (7.1)	19 (6.2)	41 (13.5)		19 (14.0)	
**Pathological HDL (%)**	231 (27.9)	76 (24.8)	58 (19.0)		23 (16.8)	
**Hypertension (%)**	68 (8.2)	28 (9.1)	112 (36.5)		44 (28.2)	
**Metabolic Syndrome (%)**	86 (10.4)	31 (10.1)	63 (20.7)		24 (18.2)	
**AFC (median [IQR])**	14.0 [8.0, 20.8]	13.0 [8.0, 21.0]	2.0 [1.0, 7.0]		2.0 [1.00, 6.00]	
**AMH (median [IQR])**	3.5 [1.6, 6.3]	3.4 [1.6, 6.1]	0.1 [0.0, 1.1]		0.0 [0.0, 0.5]	
**IL-6 (median [IQR])**	1.7 [1.0, 2.9]	1.6 [0.9, 2.5]	1.9 [1.2, 3.2]		1.5 [0.9, 3.1]	
**TNF-α (median [IQR])**	0.3 [0.2, 0.5]	0.3 [0.2, 0.5]	0.2 [0.2, 0.4]		0.3 [0.2, 0.5]	
**CRP (median [IQR])**	1.7 [0.7–4.6]	1.7 [0.7, 4.3]	1.8 [0.7, 3.9]		1.4 [0.8, 3.7]	

BMI: Body Mass Index

AMH: Anti-Müllerian Hormone

IL-6: Interleukin-6

TNF-α: Tumor Necrosis Factor-alpha

### Excluded from the analysis (*n* = 512)

The demographic and clinical characteristics of the population excluded from the analysis were largely comparable to those retained in the longitudinal study, except for mean age and educational level. (Supplemental file 1).

### Cross-sectional analysis at baseline (*n* = 829)

**Fig. 2:**
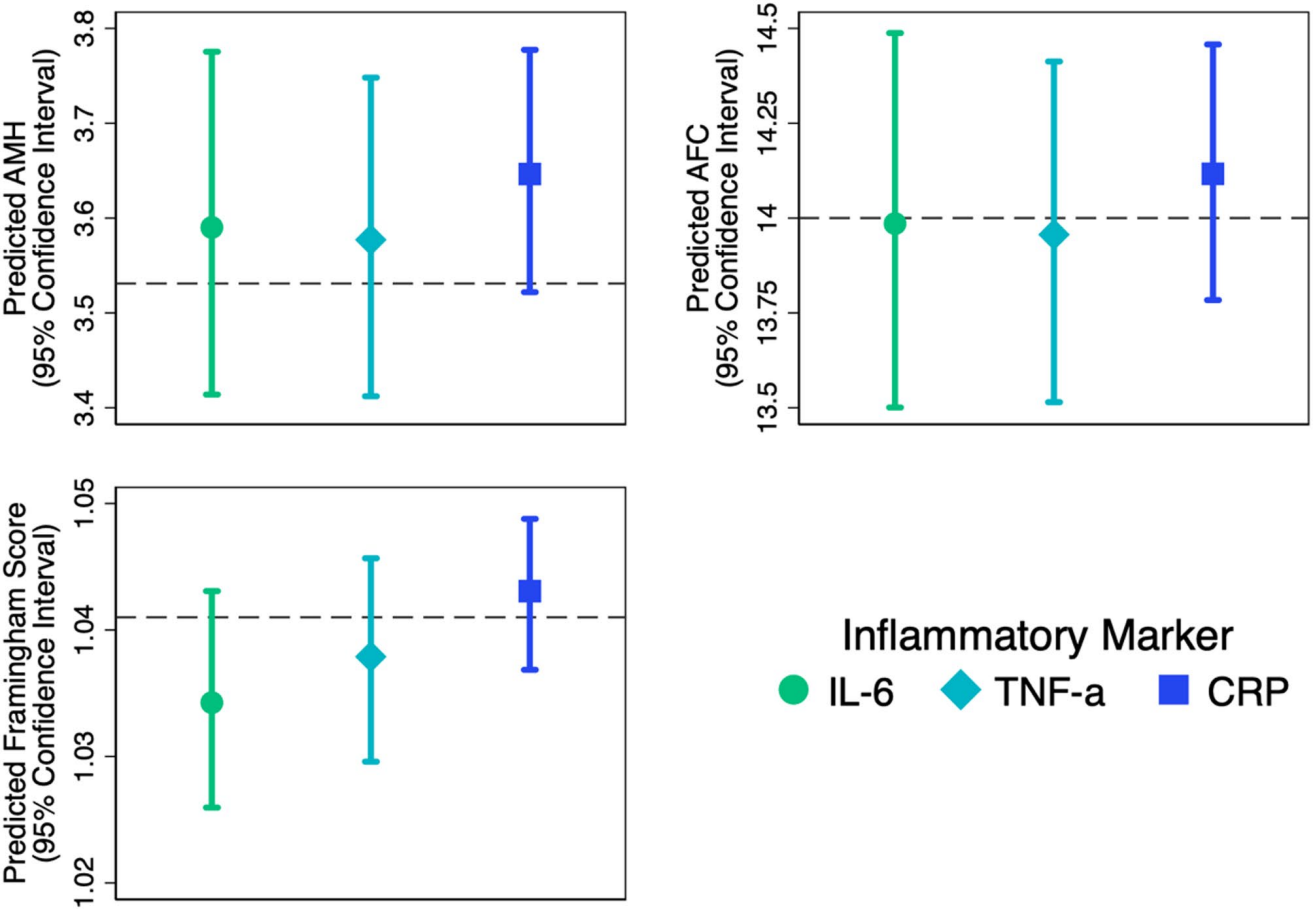
Associations between systemic inflammatory markers and ovarian aging and cardiovascular risk at baseline. AFC: Antral Follicle Count. AMH: Anti-Müllerian Hormone. IL-6: Interleukin-6. TNF-α: Tumor Necrosis Factor-alpha

The analyses revealed no significant associations between AMH levels and IL-6, TNF-alpha, or CRP (Fig. 2). Compared to the average AMH level of 3.5 ng/mL in the overall cohort, AMH levels only differed by -0.007 ng/mL (95% CI: -0.015 to 0.002) for each SD increase in IL-6 levels, -0.003 ng/mL (95% CI: -0.011 to 0.005) for each SD increase in TNF-alpha, and 0.002 ng/mL (95% CI: -0.004 to 0.008) for each SD increase in CRP. AMH levels different between those with and without metabolic syndrome, with a mean difference of 0.085 (95% CI: 0.035 to 0.134).

Similarly, no significant associations were observed between AFC and IL-6, TNF-alpha, CRP, or metabolic syndrome (Fig. 2). Compared to the average AFC levels in the overall cohort of 14.0, AFC differed by -0.059 (95% CI: -0.117 to 0.244) for each SD increase in IL-6, 0.046 (95% CI: -0.119 to 0.217) for each SD increase in TNF-alpha, 0.115 (95% CI: -0.009 to 0.246) for each SD increase in CRP, and 0.174 (95% CI: -0.646 to 1.226) for those with versus without metabolic syndrome (Table 2).

For the Framingham Risk Score, baseline inflammation levels and metabolic syndrome were not significantly associated with the score (difference in score per each SD increase in IL-6: -0.015, 95% CI: -0.499 to 0.488; TNF-alpha: -0.044, 95% CI: -0.485 to 0.413; CRP: 0.117, 95% CI: -0.216 to 0.458; metabolic syndrome 1.015;95% CI: -1.383 to 3.869) (Fig. 2).

**Table 2:** Associations between inflammatory biomarkers (standard deviation increases in IL-6, TNF-alpha, CRP, and metabolic syndrome) and ovarian aging markers (AMH, AFC) and Framingham score– Cross-sectional analysis at baseline (*n* = 829)

	Inflammatory markers	Model	Estimate^a^	95% CI Lower	95% CI Upper
AMH	IL-6	Age/BMI Adjusted	-0.084	-0.154	-0.015
AMH	IL-6	Fully adjusted	-0.007	-0.015	0.002
AMH	TNF- α	Age/BMI Adjusted	-0.027	-0.093	0.039
AMH	TNF- α	Fully adjusted	-0.003	-0.011	0.005
AMH	CRP	Age/BMI Adjusted	-0.033	-0.081	0.014
AMH	CRP	Fully adjusted	0.002	-0.004	0.008
AMH	Metabolic syndrome	Age/BMI Adjusted	-0.084	-0.469	0.301
AMH	Metabolic syndrome	Fully adjusted	0.085	0.035	0.134
AFC	IL-6	Age/BMI Adjusted	0.048	-0.128	0.233
AFC	IL-6	Fully adjusted	0.059	-0.117	0.244
AFC	TNF- α	Age/BMI Adjusted	0.057	-0.108	0.229
AFC	TNF- α	Fully adjusted	0.046	-0.119	0.217
AFC	CRP	Age/BMI Adjusted	0.072	-0.049	0.198
AFC	CRP	Fully adjusted	0.115	-0.009	0.246
AFC	Metabolic syndrome	Age/BMI Adjusted	-0.021	-0.773	0.931
AFC	Metabolic syndrome	Fully adjusted	0.174	-0.646	1.226
Framingham score	IL-6	Age/BMI Adjusted	-0.087	-0.562	0.405
Framingham score	IL-6	Fully adjusted	-0.015	-0.499	0.488
Framingham score	TNF- α	Age/BMI Adjusted	-0.058	-0.492	0.390
Framingham score	TNF- α	Fully adjusted	-0.044	-0.485	0.413
Framingham score	CRP	Age/BMI Adjusted	0.109	-0.209	0.443
Framingham score	CRP	Fully adjusted	0.117	-0.216	0.458
Framingham score	Metabolic syndrome	Age/BMI Adjusted	0.412	-1.780	2.997
Framingham score	Metabolic syndrome	Fully adjusted	1.015	-1.383	3.869

All models are adjusted for Body mass index, age, race, income, educational level, smoking status, parity, and menarche age

a: Estimates represent the difference in AMH or AFC levels and Framingham scores associated with a standard deviation increase in inflammatory markers (IL-6, TNF-α, and CRP). For the metabolic syndrome, estimates reflect the difference in AMH or AFC levels and Framingham scores between women with and without the syndrome

IL-6: Interleukin-6. IL-6 is log-transformed

TNF-α: Tumor Necrosis Factor-alpha. TNF-α is log-transformed

AMH: Anti-Müllerian Hormone

AFC: Antral Follicle Count

Log are naturals logarithms

### Longitudinal analysis (*n* = 307)

**Fig. 3:**
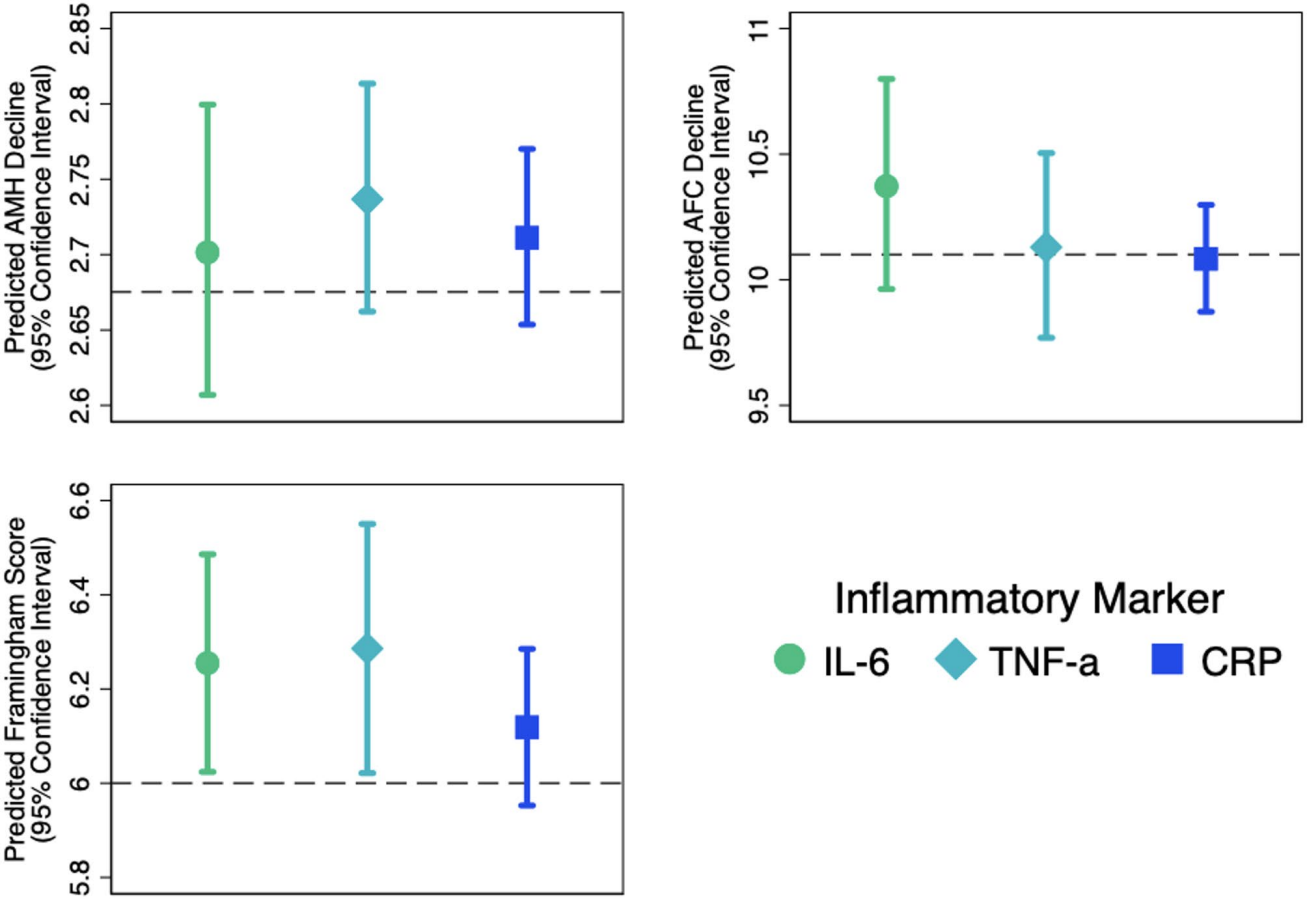
Associations between baseline inflammatory markers and longitudinal changes in ovarian aging and cardiovascular risk. AFC: Antral Follicle Count. AMH: Anti-Müllerian Hormone. IL-6: Interleukin-6. TNF-α: Tumor Necrosis Factor-alpha

On average, women in our cohort experienced a decline of 2.675 ng/mL in AMH over an 11.82-year follow-up period. After adjusting for follow-up time and baseline covariates, IL-6 levels at baseline were not significantly associated with AMH changes (Fig. 3), with an additional decline of 0.026 ng/mL (95% CI: -0.068to 0.124) per SD increase in IL-6 levels. Similarly, no significant associations were found between TNF-alpha and AMH (additional decline per SD increase in TNF-alpha: 0.061 ng/mL, 95% CI: -0.013 to 0.138) or between CRP and AMH (additional decline per SD increase in CRP: 0.036 ng/mL, 95% CI: -0.022 to 0.095) (Fig. 3). Metabolic syndrome was also not significantly associated with changes in AMH (Fig. 3), with an additional decline of 0.117 ng/mL (95% CI: -0.198 to 0.472) for those with versus without metabolic syndrome (Table 3).

Regarding AFC, women in our cohort showed an average decline of 10.100 follicles over a 12.36-year follow-up period. After adjusting for follow-up time and baseline covariates, none of the inflammatory markers were significantly associated with changes in AFC (Fig. 3; Table 3).

Women in our cohort had an average Framingham score of 6.000 after an average of 12.43 years after baseline. Significant associations were identified between IL-6 and TNF-a (scores were higher per SD increase in each inflammatory marker, by 0.255 [95% CI: 0.024–0.486] and 0.286 [95% CI: 0.022–0.550], respectively). No significant associations were identified between CRP levels at baseline or metabolic syndrome (scores were higher by 0.119 (95% CI: -0.047-0.285) (Fig. 3; Table 3).

**Table 3:** Associations between inflammatory biomarkers (standard deviation increases in IL-6, TNF-alpha, CRP), metabolic syndrome, changes in ovarian aging markers (AMH, AFC) and Framingham scores over Follow-up– GEE linear regression analysis

	Inflammatory markers	Model	Estimate^a^	95% CI Lower	95% CI Upper
AMH Decline	IL-6	Age/BMI Adjusted	0.075	-0.185	0.363
AMH Decline	IL-6	Fully adjusted	0.026	-0.068	0.124
AMH Decline	TNF- α	Age/BMI Adjusted	-0.064	-0.274	0.165
AMH Decline	TNF- α	Fully adjusted	0.061	-0.013	0.138
AMH Decline	CRP	Age/BMI Adjusted	0.111	-0.079	0.316
AMH Decline	CRP	Fully adjusted	0.036	-0.022	0.095
AMH Decline	Metabolic syndrome	Age/BMI Adjusted	-0.236	-1.300	1.652
AMH Decline	Metabolic syndrome	Fully adjusted	0.117	-0.198	0.472
AFC Decline	IL-6	Age/BMI Adjusted	-0.030	-0.802	0.806
AFC Decline	IL-6	Fully adjusted	0.273	-0.137	0.699
AFC Decline	TNF- α	Age/BMI Adjusted	-0.983	-1.633	-0.283
AFC Decline	TNF- α	Fully adjusted	0.030	-0.331	0.404
AFC Decline	CRP	Age/BMI Adjusted	0.309	-0.247	0.897
AFC Decline	CRP	Fully adjusted	-0.017	-0.227	0.198
AFC Decline	Metabolic syndrome	Age/BMI Adjusted	1.191	-1.872	5.395
AFC Decline	Metabolic syndrome	Fully adjusted	0.808	-0.351	2.105
Framingham score	IL-6	Age/BMI Adjusted	0.195	-0.066	0.455
Framingham score	IL-6	Fully adjusted	0.255	0.024	0.486
Framingham score	TNF- α	Age/BMI Adjusted	0.180	-0.129	0.489
Framingham score	TNF- α	Fully adjusted	0.286	0.022	0.550
Framingham score	CRP	Age/BMI Adjusted	0.111	-0.068	0.289
Framingham score	CRP	Fully adjusted	0.119	-0.047	0.285
Framingham score	Metabolic syndrome	Age/BMI Adjusted	1.295	-0.380	2.970
Framingham score	Metabolic syndrome	Fully adjusted	1.018	-0.510	2.547

All models are adjusted for Body mass index, age, race, income, educational level, smoking status, parity, and menarche age

aEstimates reflect the difference in AMH or AFC decline or Framingham score at follow-up for a standard deviation increase in inflammatory marker or for those with versus without metabolic syndrome at baseline

IL-6: Interleukin-6. IL-6 is log-transformed

TNF-α: Tumor Necrosis Factor-alpha. TNF-α is log-transformed

AMH: Anti-Müllerian Hormone

AFC: Antral Follicle Count

Sample size calculations for the range of effect sizes observed in these longitudinal analyses revealed that detecting a statistically significant association with 80% power would require two to three times the available sample size.

## Discussion

Inflammation has been associated with ovarian aging and thought to play a primary role in the development of CVD [4–7]. As such, we investigated markers of inflammation to support a primary role along the causal pathway between ovarian aging and the development of CVD. Our study represents one of the first investigations to simultaneously examine the cross-sectional and longitudinal associations between inflammatory markers, metabolic syndrome, ovarian aging, and cardiovascular risk. Despite existing theories suggesting a strong link between chronic inflammation and these conditions, our research did not identify significant associations in either type of analysis. As the baseline analysis was cross-sectional in design, the lack of temporality limits the ability to infer causal relationships between inflammation, ovarian aging, and cardiovascular risk. This limitation underscores the rationale for conducting complementary longitudinal analyses within the OVA.CV cohort.

The concept of inflamm-aging [35] has been implicated as a pivotal factor in both ovarian aging [9, 12, 36] and cardiovascular risk [4, 5, 7, 37]with clinical studies indicating elevated serum IL-6 levels in patients with premature ovarian insufficiency compared to their healthy counterparts [38]. This observation is also reflected in cardiovascular pathology, suggesting a potential intersection of inflammatory processes with these conditions. Given the overarching goal of the OVA.CV study to determine whether ovarian aging (prior to loss of estrogen with menopause) could serve as an early marker of cardiovascular risk, our investigation aimed to explore whether systemic inflammation represents the a common underlying pathophysiology. Although systemic markers such as IL-6, TNF-α, and hsCRP are appropriate for assessing inflammation in large-scale epidemiologic studies, they may not reflect localized intra-ovarian inflammation. Future research could benefit from evaluating markers in follicular fluid or ovarian tissue to better characterize local inflammatory processes. Given the loss of ovarian function occurs at an age years prior to onset of CVD in women, ovarian aging could serve as an early opportunity to identify women at risk of subsequent CVD and events. By identifying inflammation-associated biomarkers linked to ovarian aging, we aimed to provide insights into potential underlying mechanisms and early detection strategies for individuals at higher cardiovascular risk. In selecting IL-6, TNF-alpha, and hsCRP for our study—markers traditionally associated with chronic inflammation [39]—we hypothesized these biomarkers, along with metabolic syndrome [17] as an inflammation-associated condition, could be instrumental in the underpinnings of both ovarian and cardiometabolic decline. However, the absence of a significant association in our findings might indicate that these markers are either not specific enough or that their relevance may vary depending on the population’s age and the inter-visit interval duration. The absence of significant associations may reflect limitations in the sensitivity or specificity of the biomarkers used, or the possibility that systemic inflammation does not capture key intra-ovarian processes. In addition, the timing of biomarker assessment may not have aligned with key biological transitions, limiting our ability to capture dynamic changes in inflammatory status over time. The absence of intermediate measurements further constrains the interpretation of temporal patterns. These patterns are also illustrated in Figs. 2 and 3, where predicted values and confidence intervals show no consistent associations between inflammatory markers and ovarian aging or cardiovascular risk.

Our study reveals disparities compared to previous findings, notably an absence of association, which was also observed in a significant meta-analysis, between IL-6, TNF-alpha levels, and atherosclerotic CVD in women with PCOS [40]. Furthermore, although IL-6 has been identified as a strong predictor of future cardiovascular events [41]the relationship between TNF-alpha and normal ovarian aging remains unclear. In fact, lower levels of TNF-alpha are associated with premature ovarian failure, which may seem contradictory [42, 43]. CRP is also recognized as one of the most significant markers in the development of CVD [44] and metabolic syndrome [16]. While CRP is recognized for its association with PCOS [45]its relationship with normal ovarian aging remains ambiguous [46]. Further studies should explore additional biomarkers such as IL-8, si-CAM or PGE2 to more fully capture the complexity of the processes involved.

Our findings question the association between inflammation and ovarian aging as a common pathophysiology linking ovarian aging to CVD risk. They suggest that while inflammaging may contribute to these conditions, it does not appear to be the predominant pathway. As the frontiers of fundamental science continue to advance, revealing novel mechanisms, a challenge emerges in translating this knowledge into clinical practice and identifying early markers of CVD risk in women.

A notable limitation of our research is the substantial attrition rate for in-person follow-up visits, which was further complicated by the COVID-19 pandemic restrictions and population dynamics. Reassuringly, the demographic and clinical characteristics of those lost to follow-up did not significantly differ from participants who remained in the study, providing reassurance regarding the representativeness of our findings. However, due to this attrition, power was limited to detect significant associations given the observed effect sizes in the longitudinal analyses. At least two to three times the sample size would be required for these small effect sizes to reach a significance level of 0.05 with 80% power. Regardless of statistical significance, the differences observed remain clinically insignificant.

To our knowledge, our study is at the forefront of clinical research exploring the intricate relationship between inflammation, ovarian aging, and CVD. It represents the most extensive cross-sectional analysis in this domain to date and marks a pioneering effort as the first longitudinal cohort study in this field. The most significant strengths of our research are its large baseline population, the racial/ethnic diversity, and the extended duration, including a planned follow-up visit.

Our rigorous inclusion criteria, particularly the exclusion of PCOS patients, reduced the potential bias related to chronic inflammation. However, these strict criteria may have also excluded older women with accelerated aging, affecting both those with high ovarian reserve (PCOS) and low reserve. Despite this, the broad range of ovarian aging parameters ensures that our results are widely applicable to most of the female population.

The combination of cross-sectional and longitudinal analyses in our study offers a comprehensive view [18]bridging the gap between the immediate state and the evolution of the conditions over time. This dual approach provides a deeper and more nuanced understanding. Continued follow-up may allow association with clinical disease.

## Conclusion

Our study adds to the debate on the links between inflammation, ovarian aging, and cardiovascular risk. By showing no association in a large cohort, we emphasize the need for more detailed research on chronic inflammation biomarkers and their role in women’s health.

## Electronic supplementary material

Below is the link to the electronic supplementary material.

Supplementary Material 1

## Data Availability

The datasets analyzed during the current study are not publicly available due to participant confidentiality and institutional restrictions. Data may be made available from the corresponding author on reasonable request and with appropriate institutional approvals.

## Declarartion

### Data access and responsibility

Dr. Marcelle Cedars had full access to all the data in the study and takes responsibility for the integrity of the data and the accuracy of the data analysis.

## Abbreviations

AFC: Antral Follicle Count
AMH: Anti-Müllerian Hormone
BMI: Body Mass Index
CVD: Cardiovascular Disease
CRP: C-Reactive Protein
ELISA: Enzyme-Linked Immunosorbent Assay
GEE: Generalized Estimating Equation
HDL: High-Density Lipoprotein
hsCRP: High-Sensitivity C-Reactive Protein
IL-6: Interleukin-6
KP: Kaiser Permanente
OVA / OVA.CV: Ovarian Aging Study / Ovarian Aging and Cardiovascular Risk Study
PCOS: Polycystic Ovary Syndrome
SD: Standard Deviation
TNF-α: Tumor Necrosis Factor-alpha
TVUS: Transvaginal Ultrasound
