# Supplementary material for: Ovarian aging, cardiovascular risk and inflammation: insights from the OVA study
Source: J Ovarian Res. 2025 Jul 26;18:164. doi: 10.1186/s13048-025-01754-8 (PMC12297770; doi:10.1186/s13048-025-01754-8)
Supplement: Supplementary file 1 — Supplementary Material 1 [file 13048_2025_1754_MOESM1_ESM.docx]

**Supplemental 1: Comparison of the excluded Population and the Follow-Up Population**

|  | **Excluded** | **Follow-Up** | **p-value** |
| --- | --- | --- | --- |
|  | 512 | 317 |  |
| **Age (mean (SD))** | 34.9 (5.5) | 35.8 (5.4) | 0.021 |
| **BMI (mean (SD))** | 27.5 (6.8) | 27.4 (7.2) | 0.857 |
| **Waist Circumference (median [IQR])** | 80.6 [72.9, 93.2] | 80.3 [72.4, 92.3] | 0.571 |
| **Nulliparity (%)** | 298 (58.2) | 178 (56.2) | 0.251 |
| **Race (%)** |  |  |  |
| Caucasian | 151 (29.5) | 102 (32.2) | 0.389 |
| African American | 129 (25.2) | 72 (22.7) |  |
| Chinese | 85 (16.6) | 64 (20.2) |  |
| Filipino | 23 (4.5) | 9 (2.8) |  |
| Latina | 124 (24.2) | 70 (22.1) |  |
| **Income (%)** |  |  |  |
| Less than $5,000 | 3 (0.6) | 5 (1.6) | 0.154 |
| $5,000 through $11,999 | 11 (2.2) | 6 (1.9) |  |
| $12,000 through $15,999 | 14 (2.8) | 7 (2.2) |  |
| $16,000 through $24,999 | 34 (6.7) | 17 (5.4) |  |
| $25,000 through $34,999 | 73 (14.3) | 32 (10.2) |  |
| $35,000 through $49,999 | 112 (22.0) | 69 (21.9) |  |
| $50,000 through $74,999 | 93 (18.3) | 83 (26.3) |  |
| $75,000 through $99,999 | 64 (12.6) | 38 (12.1) |  |
| $100,000 and greater | 105 (20.6) | 58 (18.4) |  |
| **Education Level (%)** |  |  |  |
| Less than high school / Some high school | 48 (9.4) | 19 (6.0) | 0.049 |
| High school graduate/GED | 56 (10.9) | 26 (8.2) |  |
| Some college (AA) /Vocational/technical school | 120 (23.4) | 88 (27.8 |  |
| College graduate (bachelor’s degree) | 197 (38.5) | 110 (34.7) |  |
| Graduate school (Doctorate, Master’s degree)/ Professional school (MD, JD, DDS, MBA) | 91 (17.8) | 74 (23.3) |  |
| **Smoking Status (%)** | 133 (26.0) | 74 (23.3) | 0.442 |
| **Pathological Triglycerides (%)** | 66 (12.9) | 27 (8.5) | 0.068 |
| **Pathological Fasting Glucose (%)** | 39 (7.6) | 20 (6.3) | 0.567 |
| **Pathological HDL (%)** | 151 (29.5) | 80 (25.2) | 0.212 |
| **Hypertension (%)** | 40 (7.8) | 28 (8.8) | 0.697 |
| **Metabolic Syndrome (%)** | 54 (10.5) | 32 (10.1) | 0.928 |
| **AFC (median [IQR])** | 14.0 [8.0, 20.3] | 13.0 [8.0, 20.8] | 0.543 |
| **AMH (median [IQR])** | 3.7 [1.7, 6.6] | 3.4 [1.6, 5.8] | 0.372 |
| **IL-6 (median [IQR])** | 1.7 [1.0, 3.1] | 1.6 [0.9, 2.5] | 0.052 |
| **TNF-α (median [IQR])** | 0.3 [0.2, 0.5] | 0.3 [0.2, 0.5] | 0.651 |
| **CRP (median [IQR])** | 1.7 [0.7, 4.8] | 1.7 [0.7, 4.2] | 0.139 |

BMI: Body Mass Index.

AFC: Antral Follicle Count

AMH: Anti-Müllerian Hormone.

IL-6: Interleukin-6.

TNF-α: Tumor Necrosis Factor-alpha.
